# Public Health Achievements in Rwanda: A 21st Century Transformation

**DOI:** 10.1002/puh2.70225

**Published:** 2026-04-21

**Authors:** Niyibizi Julius, Mugisha John, Nizeyimana Emmanuel

**Affiliations:** ^1^ Southern Medical University Guangzhou China

**Keywords:** community health workers, infectious, Rwanda public health, universal health coverage

## Abstract

This narrative review documents how Rwanda has transformed in public health extraordinarily post the 1994 Genocide against the Tutsi, placing it as an exemplary model for health care systems resilience in low‐income countries. The review is based on the World Health Organization building blocks to look at the strategic changes that have led to measurable gains in the health of Rwandan people. Good centralized leadership and control have been key to this accomplishment. This made it possible to decentralize service delivery, build excellent health information systems, and invest money in the health workforce. A key part of the transformation is universal health coverage (UHC), especially mutuelle de santé, which increased coverage from 27% in 2004 to more than 85%, hence cutting down out of pocket costs, which improved equity. Integration and use of community health workers (CHWs) have been instrumental in expansion of primary care to the population mostly in rural settings, improving maternal and child life, tuberculosis (TB) treatment through direct observed therapy (DOT), and disease surveillance. These coordinated actions have resulted in substantial reductions in mortality from infectious diseases (HIV/AIDS, TB, and malaria), maternal and child health indicators, and the gradual integration of services for noncommunicable diseases and mental health. Rwanda's health system was stress tested and proved its effectiveness in the COVID‐19 and Marburg outbreaks, proving exceptional planning and rapid response capacities. Despite Rwanda's achievement, obstacles still persist, such as reliance on foreign funds, limited human resources lowering the quality‐of‐service delivery, and mental health challenges still in existence. Rwanda's experience illustrates that a proactive government, citizen participation, and research‐based innovation may produce rapid and significant overall health gains, providing a valuable model for similar situations to other countries.

## Graphical abstract

This narrative review examines Rwanda's post‐2000 public health transformation using the WHO health system building blocks. It shows how strategic governance, universal health coverage via mutuelle de santé, community health workers, and digital health tools have driven substantial gains in maternal/child health, infectious disease control, and outbreak resilience. Rwanda's experience provides a replicable model for strengthening health systems in low‐resource settings, particularly by demonstrating how integrated approaches can improve access to care and health outcomes for vulnerable populations.

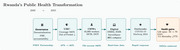

## 1 Introduction

The transformation that Rwanda has made to become a global inspiration for progress in public health since 2000 represents an unparalleled example of health system resilience and innovation. Recovering from the 1994 genocide against Tusti that destroyed the country, including half of its population, infrastructure, and economy, Rwanda had tremendous obstacles ahead, such as reducing high mortality rates, for which access to health services was lacking and the health system was not coherent. This article analyzes Rwanda's public health transformation since 2000 utilizing the World Health Organization (WHO) health system building blocks. It illustrates how strategic reforms across these domains have driven measurable improvements in population health outcomes.

## 2 Methodology

This narrative review synthesized the evidence base for the public health transformation in Rwanda post‐2000, framed with in the WHO system building blocks of: service delivery, health workforce, information systems, medical products, vaccines and technologies, financing, and leadership/governance. This review offers a descriptive overview of significant achievements and challenges, as well as lessons learned, based upon a selection of peer‐reviewed literature, policy documents, and reports sifted through an expert‐guided review.

## 3 Literature Identification and Selection

Targeted searches were done in electronic databases such as PubMed, Scopus, the Web of Science, and Google Scholar, as well as Africa's equivalent—WHO African Index Medicus (AIM)—to include both global and regional views. Manual searches on gray literature such as the Rwanda Ministry of Health website, the World Bank Open Knowledge Repository, and UNICEF reports for policy documents and national health surveys were also conducted. The review concentrated on content published from January 2000 to September 2025 that reflects the post‐genocide recovery period that also includes contemporary events such as the COVID‐19 coronavirus pandemic and the Marburg outbreak in 2024.

Search terms were iteratively refined and included combinations such as (“Rwanda” OR “Rwandan”) AND (“public health” OR “health system” OR “health care reform” OR “universal health coverage” OR “mutuelle de santé” OR “community health workers” OR “maternal child health” OR “communicable diseases” OR “noncommunicable diseases” OR “mental health” OR “digital health” OR “health financing” OR “governance”). Boolean operators (AND, OR) and truncation (e.g., “health*”) facilitated broader retrieval. English‐language publications were prioritized due to accessibility, with no initial language restrictions.

Inclusion criteria encompassed sources that (1) addressed Rwanda's public health system or its components (e.g., universal health coverage [UHC], disease control, and workforce development) post‐2000; (2) presented empirical data, policy analyses, or evaluations of health outcomes and interventions; and (3) comprised peer‐reviewed journal articles, systematic reviews, or official reports from credible organizations (e.g., WHO and World Bank). Materials were excluded if they (1) lacked specificity to Rwanda; (2) predated 2000; (3) consisted solely of non‐empirical opinion pieces, editorials, or abstracts without substantive data; or (4) represented duplicates.

## 4 Data Extraction and Synthesis

Relevant studies were analyzed, and data were extracted in a structured format. This included the study outline, population of focus, health outcome and intervention findings, and positions per building block of the WHO, as well as scope and limitations. Relevance of the data extracted was to the overriding theme of the study.

A narrative approach to data integration was used, combining findings longitudinally and thematically as structured by the WHO building blocks and influenced by contextual nuances. Principal emerging ideas, for example, governance shifts, expansion of UHC, and emergency outbreak response, were documented alongside relevant quantitative measures, for example, mortality and service coverage. Both the absence of an evidence synthesis approach and the interpretative nature of the review were used to justify the absence of a formal methodology to study quality. This flexibility allowed for an articulated account of the health system within a context of clear source selection and integration.

## 5 Evolution of Rwanda's Health Sector

Before the 1994 Genocide against the Tutsi, Rwanda's health system comprised 34 hospitals, 186 health centers, one blood bank, and fewer than 300 doctors. Much of this infrastructure was destroyed during the genocide, and the health workforce was severely depleted. By 2012 it had built 440 health centers and 42 district hospitals covering 85% of the populations [1]. These later expanded to 520 health centers, 1179 health posts [2], and an upgrade leading to 34 district hospitals, 6 level 2 teaching hospitals, 9 teaching and referral hospitals, and 5 specialized hospitals [3]. By 2011, the mortality rates were reduced by 77.1% and health care providers to populations were 1.9/10,000 [4].

Throughout the 21st century, Rwanda's health system has experienced a radical transformation through the pursuit of structural reform and efforts driven toward policy convergence. A portion of this early work included the recovery of post‐genocide country infrastructure and the development of confidence in public institutions that enabled later reforms of the sector [5, 6]. Through a decades‐long political campaign to decentralize health services, including the Population Health Implementation and Training (PHIT) Partnership, access and service quality have significantly improved, with district‐level integration enhancing care delivery [5, 7]. Health system reinforcement has been an important contributory factor for achieving the Millennium Development Goals (MDGs), particularly for maternal and child health results [5, 6]. A review of policy reforms and usage of health services highlighted that community‐based strategies and strong clinical practices support the entire cascade of care [8].

Harnessing sustainability, Rwanda invested in building the capacity of its health workforce and promoting sound governance frameworks as enablers for accountability and continuous improvement. The governance analysis revealed that the commitment to health sector reform was a joint initiative of the national government and international agencies, which provided a conducive atmosphere for new public health innovations to develop [5]. Notably, the establishment of a National Public Health Institute and the implementation of the indicator‐based surveillance (IBS) system, with 96.7% completeness and 80.8% timeliness, have centralized outbreak responses and harmonized public health policies, strengthening evidence‐based decision‐making [9]. Gradually, the application of evidence‐based practices and long‐term strategic planning has additionally supported the outstanding performance that Rwanda has made in the transformation of the sector into one of the sub‐Saharan Africa's best [5, 6].

### 5.1 Health System Strengthening and Governance

The systematic building of the health system, along with improved governance, is at the heart of Rwanda's public health success. This included reform of policies, construction of infrastructure, and development of stringent monitoring systems to guarantee compliance with health standards [5, 6]. However, enhanced governance coupled with selective donor investment led to scale reforms across the board that linked primary care with a range of services up to and including specialized care. This is reflected in the strong referral systems that have been established between community, district, and tertiary services [8].

In addition, the health governance reforms created an institutional context conducive to the work of local health authorities. Bottom‐up approach to implement policy. Decentralization policy, initiated in 2000, established integrated health management teams at district and sector levels. These teams, comprising the District Director of Health, hospital directors, planning officers, and representatives from health centers, community health workers (CHWs), and stakeholders, operate under the Vice Mayor in charge of Social Affairs. They oversee district‐wide planning, resource allocation, supervision, and accountability through *imihigo* performance contracts. This bottom‐up approach has strengthened local ownership and service integration [5]. Advanced digital health solutions, including electronic medical records (EMRs) and mobile technologies, were implemented to enhance data surveillance and decision‐making, achieving high levels of data completeness and timeliness [9]. The development strategies and processes of health needs, along with political, technological, and policy changes, have been mapped to interventions and measured improvements in public health outcomes through governance reforms and technological advancements [5]. These strategic interventions not only optimized service delivery but also achieved the greatest impact of reform by reaching the most vulnerable population [5, 6].

### 5.2 Health Insurance and Universal Coverage

The initiation and scale‐up of the community‐based health insurance (CBHI “Mutuelle de Santé”) is regarded as one of the most notable achievements of Rwanda's health system. Even before these reforms, there were substantial financial barriers to health care. Health insurance coverage rose from 27% in 2004% to 74% in 2007, reaching over 85% by 2025, due to reduced out‐of‐pocket payments, significantly improving equity in health service access [10]. Currently, the scheme covers over 85% of the Rwandan population, a major step toward UHC [11].

The increase of health insurance has been linked to improved health care outcomes, particularly in rural Rwanda, where poverty previously limited access to health services, though challenges like adverse selection persist among the poorest households [10, 12]. The existing evidence suggests that maternal and child health service utilization and perinatal and under‐five mortality were directly proportional to increases in health insurance coverage [13]. Financial reforms and good strategies for health spending have enabled Rwanda to implement scale‐up of priority public health interventions well, ensuring the sustainability of public health gains [5, 13]. The CBHI model is therefore not just a successful health financing innovation, but it has also triggered other reforms on the national health agenda [5].

### 5.3 Service Delivery

#### 5.3.1 Maternal and Child Health

The maternal and child health care status in Rwanda has greatly improved since 2000. Some systematic reviews noted impressive gains in ANC coverage, SBA, and PNC services [5, 6]. Community‐wide interventions, including those driven by CHWs and supported by UNICEF and World Bank initiatives, have significantly reduced maternal and newborn mortality through increased prenatal care attendance and institutional deliveries [14]. Evidence indicates that packages of interventions that include health education, improved service delivery, and community‐based mobilization have resulted in a measurable contribution to the increase in service coverage [5, 6].

Studies suggest that these interventions resulted in remarkable reductions in maternal morbidity and adverse birth outcomes [15]. Simple preventive strategies have also been included, like integrating periodontal screening with antenatal care, which has led to better outcomes of pregnancy and a decrease in preterm birth [16]. These well‐coordinated strategies followed a comprehensive approach, which is needed to make tangible progress in maternal and child health care.

### 5.4 Control of Communicable Diseases: HIV/AIDS, Tuberculosis (TB), and Malaria

Rwanda has been successful in controlling communicable diseases which has contributed to its overall public health success. In the early 2000s, the expansion of HIV testing, counseling, and voluntary medical male circumcision programs (which have since risen to prominence in the national HIV prevention strategy) was initiated [17, 18]. Effective incorporation of programs like highly active antiretroviral therapy (HAART) and community‐level education into existing health services, in turn, has led to the adoption of healthier practices and to a decrease in the rate of transmission of HIV [14, 17, 18]. In addition, ongoing public health efforts have resulted in SB testing coverage and AG treatment seeking rates in favor of SB/AG‐associated control of the disease [18].

TB control in Rwanda has seen substantial progress through integration with general health care delivery, improving case detection rates and treatment adherence, supported by community‐based strategies [19]. In recent years, malaria control strategies, including vector control, prompt diagnosis, and integrated community case management (iCCM), have focused on endemic areas, significantly reducing transmission risk [20, 21, 22].

Analyses based on the structural equation models show the wider access to health insurance and integrated public health approaches have directly led to a decline in malaria morbidity and have generally been beneficial to childhood health in the malaria‐endemic countries [20, 21]. In combination, these interventions highlight the ability of Rwanda to harness a “convergence” approach to CCDs affordably and holistically.

### 5.5 Noncommunicable Disease Care and Prevention

NCDs, including cancer, are increasingly a priority in Rwanda's health policy landscape. The setting up of flagship cancer hospitals and adoption of national cancer control strategies has led to better screening, diagnosis, and treatment regimens [11]. Immunization campaigns, specifically for human papillomavirus (HPV) and hepatitis B, have further shared the burden of some cancers [11, 23]. The targeted approach of Rwanda in the prevention of cervical cancer has brought about an observable reduction in cervical cancer incidence and may act as an example for other sub‐Saharan countries facing the same struggle [23].

Lifestyle interventions and community‐based health education and decentralized care models, supported by public–private, have been introduced to prevent and manage NCDs, improving access to medications and diagnostic services [19]. This continuum of care in the follow‐up of complications of chronic disease, associated with the use of evidence‐based clinical protocols, resulted in better outcomes and increased survival. Collectively, these changes underscore that a deliberate and coordinated public policy supported by strong health system infrastructure can lead to substantial gains in NCD control [11, 23].

### 5.6 Psychological Health and Developmental Health Issues

Although mental health has historically been neglected in many low‐income countries, Rwanda has made impressive efforts. Acknowledging the double burden of mental health problems and traditional beliefs, Rwanda has made significant efforts to integrate mental health into its public health agenda, with specialized centers, home‐visit programs, and culturally sensitive community initiatives addressing PTSD and substance abuse [24, 25]. Some recent work has examined the intersection of traditional and biomedical health care in Rwanda, concluding that mental health care in Rwanda continues to be more evidence‐informed than before but still roots itself in culture [26]. In support of these developments, national programs have been launched to educate local volunteers and social workers to provide trauma‐informed care in support of both psychiatric and psychosocial interventions [27].

In addition, real‐time data on new health threats, like fungal infections, are also being recorded in a standardized manner to guide policy and intervention planning [28]. New digital reporting tools that accompany an overall emphasis on public health have helped fortify the capability to track and address the threat of emerging infectious diseases. Comprehensive interventions, including community‐based interventions, mobilized health workforces, and strong monitoring and evaluation systems, have strengthened mental health services and health security in Rwanda [26, 28].

### 5.7 Health Workforce

#### 5.7.1 Health Workers and CHW Strategies

Rwanda's investment in building its human resources for health directly facilitated the scale‐up and decentralization of health care. Investment in CHW programs has been central to the devolution of primary care and the extension of services to outlying and marginalized populations [5, 6]. CHWs are the first level of intervention at the community level; they deliver critical MCH services and make substantial contributions to disease surveillance and also immunization interventions [5, 6]. Simultaneously, specific training initiatives have been able to strengthen the capacity of skilled health care providers and promote a culture of ongoing quality improvement within the health facilities [5].

The organized incorporation of CHWs within the larger health system allowed for the rapid increase of health interventions and improved health results. The effectiveness of these interventions has been supported by adherence supportive of adolescents in pregnancy and birth, a strong supervision system, and incentives to encourage health workers [5] to practice best practice. Such interventions have strengthened the efficiency of service delivery, and the existence of continuous feedback systems is also conducive to the development of policies and health systems reform [5]. Among the major policy initiatives introduced have been the adoption of mobile health clinics and the constant support in the training of skilled birth attendants. Within policy frameworks, early detection and intervention programs, supported by national strategic plans, have been prioritized, though financial barriers and limited human resource capacity remain challenges [10, 11, 23]. Beyond CHWs, the government of Rwanda, together with development partners, in 2012, integrated a human resource for health program for 7 years for specifically transforming health care providers through education aimed at increasing the numbers of health care providers such as doubling medical students and a 60% increase of nurses and midwives [29, 30, 31]. Despite these gains, the density of skilled health professionals remains low at approximately 1.9 doctors, 6.1 midwives, and 11.6 nurses per 10,000 population [32], particularly affecting quality of care in rural areas. This improvement of retention leads to improving continuity of care, hence improved health outcomes.

### 5.8 Health Information System

#### 5.8.1 Digital Health Tools and Data Systems Utilization

The fast growth of the information and communications technology (ICT) industry has contributed to the transformation of the public health system in Rwanda. The implementation of digital health applications, such as the IBS system with 96.7% completeness and 80.8% timeliness, has significantly improved data collection, disease surveillance, and program monitoring, enabling rapid, evidence‐informed decision‐making at various governance levels [9].

Digital tools, in the meantime, have proved helpful in the effort of mapping health necessity and service delivery tracking, as well as guaranteeing that interventions are appropriately tailored to those most at risk [5, 33]. Moreover, health spending studies and time series data, which have been cleaned through machine learning models, are used to inform prioritization in investing resources for child health programs [13].

The ICT‐enabled transformation in Rwanda's health system is an exception not just because of its productivity impact, but also because of how it has empowered health workers and communities. The linkage of digital data systems to routine health services is a game‐changing innovation for policy‐making, allowing for ministries of health to make real‐time assessments on health and the effectiveness of interventions [13, 33]. These types of innovation have helped to earn Rwanda a mantle as a forward‐looking country concerning public health and as a model for other countries in adopting digital technology [5, 33].

Rwanda has a routine health management information system that is built on district health information system 2 (DHIS2) that collects, validates, analyzes, and visualizes health data across all systems from health centers to referral hospitals and is thereafter used at the central level for decisive actions and information sharing [34]. Rwanda also utilizes other EMRs, which were initially enrolled in 2005. These EMRs, like OpenMRS for infectious diseases like HIV/TB, community electronic records, and integrated community health information system [35, 36]. These data management systems, especially in HIV and TB, have strengthened patient care, minimized errors, and increased monitoring rates. Additionally, Rwanda pioneered merging the individual and aggregated information systems by inserting EMRs outputs into the DHIS2, establishing a more integrated an efficient health information network [34]. A study indicated that patients who live in Kigali perceived EMR use positively relating them to improved care quality and confidentiality [37]. However, recently research indicated that there are some barriers like internet connections, stuff turnover, and lack of enough training [38].

### 5.9 Access to Essential Medicines, Vaccines, and Technology

Rwanda has markedly enhanced access to essential medicines via smart supply chain reforms and financial protection measures. Rwanda's Medical Supply institution was founded in 2012 to consolidate the procurement and distribution of pharmaceuticals, significantly diminishing stock outs and guaranteeing the continuous supply of medications such as anti‐retroviral drugs, antibiotic drugs, and maternal health products to all of the country [39], and it is on the WHO model list of essential medicines as it incorporates disease burden priorities such as HIV, TB, NCDs, and mental health [40, 41]. The mPfizer manufacturing plant was recently brought to Rwanda in the special economic zones, and it will be producing mRNA vaccines.

#### 5.9.1 Immunization of Diseases in Rwanda

Immunization efforts have been key to Rwanda's public health transformation. There has been a dramatic decline in the prevalence of vaccine‐preventable diseases with the development and implementation of a wide spectrum of vaccines. Rwanda has achieved high coverage for vaccinations against measles, rubella, HPV, and hepatitis B through national‐level efforts, supported by UNICEF and other international partners, significantly reducing the burden of vaccine‐preventable diseases [11, 14, 23]. The successful implementation of nationwide vaccination efforts, involving cold chain upgrades and community mobilization, has significantly reduced the burden of morbidity and mortality among children [11].

Additionally, the use of strategies such as school‐based vaccination and outreach services in rural areas has been instrumental in sustaining high immunization uptake and overcoming physical access barriers due to the dispersed nature of the population [11]. The immunization gains are a microcosm of the wider health system changes and demonstrate the country's ability to make swift and durable actions toward public health interventions [11, 23].

These reforms, including health system strengthening, UHC through the Mutuelle de Santé program, maternal and child health improvements, control of communicable and noncommunicable diseases, mental health service integration, environmental health advancements in water, sanitation, and hygiene (WASH), and the adoption of digital health systems like the IBS system, serve as a model for low‐income countries [5, 6, 9].

Life expectancy has risen from 49.7 years in 2001 to 69.6 years in 2022, indicating strong health system reforms [42]. These reforms, including health system strengthening, UHC through the Mutuelle de Santé program, maternal and child health improvements, control of communicable and noncommunicable diseases, environmental health advancements in WASH, and the adoption of digital health systems like the IBS system, serve as a model for low‐income countries [5, 9, 10]. Mental health services decentralization and integration were expanded to local health facilities such as health centers, and mental health focal persons were introduced [43, 44].

Rwanda's success is underscored by its ability to mobilize resources from government, community, and international partners, achieving remarkable reductions in mortality and pioneering innovations in service delivery, such as the PHIT Partnership and robust digital surveillance systems [7, 9]. The significance of Rwanda's experience is that it has not only produced substantial public health gains but also showcases one of the African health reform templates, where strategic governance and evidence‐based interventions can lead to measurable improvements [6]. This article is an evidence‐based description of Rwanda's public health transformation since 2000.

## 6 Response to Emerging Outbreaks

### 6.1 COVID‐19 Response and Health Security

It was unprecedented in that it had similar impacts globally on the health system, but Rwanda's response highlighted its readiness and public health system that can withstand shocks. Comparative COVID‐19 responses show that Rwanda responded to the epidemic promptly and in a coordinated manner, focusing on early detection, wide testing, contact tracing, and community engagement [45]. The creation of emergency operational centers, supported by the country's robust digital health infrastructure, facilitated the dissemination of information and resource allocation in the pandemic [45].

The public health preparedness of Rwanda to COVID‐19 was additionally strengthened by the deployment of CHWs and nationwide public awareness, whereby COVID‐19 control measures reached even the most rural with those epidemics [45]. The rapid rollout of response methods not only kept the virus in check but also reduced other health effects, including a shortage of routine immunization services and maternal services [45]. This joint response has been widely seen as among the most successful in the region, underscoring the value of initial health system strengthening and governance reforms [5, 45].

### 6.2 Marburg Virus Preparedness and Response

In 2024, Rwanda saw its first ever Marburg virus disease outbreak case, a very virulent disease with fatality rates reaching 88%. The nation's swift and unified reaction demonstrated the robustness of its public health systems [46]. The initial Marburg case confirmation, the Ministry of Health initiated its public health emergency command center utilizing IBS for real‐time case detection, contact tracing, and risk communication [46, 47]. Isolation units were quickly set up in referral hospitals, and CHWs communicated prevention messages while monitoring potential contacts in affected districts. Rwanda's Biosafety Laboratory level 3 enabled prompt diagnostic confirmation. Rwanda effectively managed this outbreak via transparency, daily situation reports, and coordination between institutions, which built public trust and prevented panic. This indicated strong government commitments, integrated surveillance, and readiness of the front liners in addressing emerging viral threats, even in resource‐limited environments.

### 6.3 Challenges and Lessons Learned

Rwanda's advancing public health achievements are, however, being constrained by the sustainability of CBHI, emerging diseases, and scale‐up of mental health services. Key challenges include scarce resources (both domestic and external funding). Quality of service such as waiting time, disparities in settings, and quality of specialists must also be improved to help diminish the rural coverage and outcome gap. The reform of the health sector in Rwanda: the importance of good governance, community participation, and information technology. The emphasis of the policymakers should be on strategic allocation of resources, sustainable financing and to inclusion the concept of innovation in the service delivery. New threats, such as noncommunicable disease and mental health problems, require cross‐sector cooperation.

### 6.4 Comparison With Other African Countries

Rwanda has a distinctive configuration of public health in Sub‐Saharan Africa with integrated maternal and child health, decreasing rates of mortality inputs, and is a benchmark country for adoption of digital health tools, not because *t* managed to eliminate all health problems, but strived to integrate and strengthen WHO building blocks, equitably. Rwanda managed came from the worst state of Genocide against Tutsi to overcome the challenges of COVID‐19 and Marburg within rapid responses. The quick response, resilience in decision‐making and sufficient funding by the Rwandan government resulted in low morbidity and mortality, integration of CHWs, turning Genocide into a model of resilience, and serve as a model for public health innovation in the region [45]. Rwanda, same as East African countries, has made stable progress with the pace, health equity, and being coherent based on the past bitter experience it passed through.

However, a sustainable health system remains a challenge as Rwanda still relies on external funds, has fewer health care providers, and has mental health and NCDs. Rwanda demonstrates that effective health systems are built on zeal, commitment, and equity instead of abundant utilization of resources. Rwanda is on the right path to eliminate and manage evolving disease burdens and financial constraints, offering a blueprint for achieving health for all (Table 1).

**TABLE 1:** Comparison of East African Communities’ World Health Organization (WHO) building blocks using most recent Demographic Health Survey.

WHO building block and indicator	Burundi	DRC	Kenya	Rwanda	Uganda	Tanzania
**1. Service delivery**						
% of women with 4+ antenatal care visits (ANC4+)	49%	45%	66.6%	98%	57%	65%
% of births in a health facility	84%	83%	88%	93%	73%	81% live birth and 93% still birth
% of basic vaccination (12–23 months)	85%	21%	80%	96%	55%	53%
% of children u5 with fever who received antimalarial treatment	69%	51%	42%	62%	87%	75%
**2. Health workforce**						
Estimated skilled health workers per 1000 population	≤1	From 1 to 2.2	From 1 to 2.2	≤1	≤1	≤1
CHW coverage	11,845 CHWs	—	58,079 (CHVs)	45,000	179,000 (CHVs)	0
**3. Health information systems (HIS)**						
National use of DHIS2	Addressing challenges to implementation of DHIS2	In the buildup of DHIS2 capacity	Fully implemented with dashboards	Strong performance with real‐time data use	Successfully implemented	Becoming active and expanding
U5 birth registration	84%	34%	76%	86%	40.4%	68%
**4. Access to essential medicines & technologies**						
% of modern contraceptive use	23%	11%	57%	35.1%	38%	31%
% of households with at least one ITN	63%	73%	73%	88%	84%	
% of ITNs used the night before survey	46%	69%	57%	66%	83%	74%
**5. Health financing**						
Health insurance coverage (15–59 years)	21.8%	4%	26%	83%	4%	<10%
Serious challenges faced by women to health care access at health facility (HF)	‐ Distance to HF ‐ Lack of finances	Distance to HF ‐ Lack of finances ‐ Permission to visit HF	‐ Distance to HF ‐ Lack of finances	‐ Distance to HF ‐ Lack of finances		‐ Distance to HF ‐ Lack of finances
Government health expenditure per capita (USD)	8 (12%)	5 (4.1%)	56 (4.7%)	29 (6.2%)	9 (4.3%)	24 (3.8%)
**6. Leadership and governance**						
Health system structure	Provincial health directorates, district manages, and central level sets laws	3 tier, decentralized, and health zone model	Counties manage primary and secondary care, central manages national hospitals	Decentralized system and highly centralized, via PBF, CBHI	Decentralized; policy‐practice gaps	Decentralized by devolution, mainly owned by councils
Key governance strength	Strong primary care policy alignment	Interventions to boost health services	Devolved systems, high digital uptake	Accountability, performance contracts, UHC, results‐based financing	Strong malaria/HIV programs	Political stability, e‐government platform, decentralized system
Major governance challenge	Corruption, finances	Conflict, corruption, aid dependence, finances	Equity in devolved system, corruption, debts, finances	Aid dependency, finances	Corruption, finances	Finances

Abbreviations: CBHI, community‐based health insurance; CHWs, community health workers; DHIS2, district health information system 2; UHC, universal health coverage.^47^

## 7 Conclusion

Rwanda's public health gains since 2000 are one of the most impressive stories of success in sub‐Saharan Africa. Through extensive health system reform, CBHI, Rwanda has achieved considerable gains to fight against all communicable and noncommunicable diseases, and investment in digital health, it has made considerable gains not only in terms of better health outcomes but also of great adaptability to emerging threats such as COVID‐19. CHW strategies are integrated well, the use of ICT in the delivery of health services is extensive and the political commitment to reforms has been maintained.

Challenges persist, such as resource generation, quality of services, and the expansion of mental health services but Rwanda's experience with evidence‐based policy‐making, with good governance and the engagement of communities, is a reflection on the potential to achieve rapid gains in public health. The achievement of the country holds lessons for many other African countries and reinforces the need for relentless innovative and adaptable public health tactics. As Rwanda continues to improve its process, the experiences reported over the last two decades offer a solid framework on which to base continued improvements, which can serve as an example of successful models for similar settings elsewhere in the world.

## Author Contributions

Niyibizi Julius and Mugisha Joh conceived the initial research idea. Nizeyimana Emmanuel performed the systematic search, screened the retrieved studies based on key words, titles and abstracts. Mugisha John screened the full texts. Mugisha John extracted the data. Niyibizi Julius wrote the first draft of the article. Nizeyimana Emmanuel wrote the abstract. All authors discussed the results and commented on the manuscript. Niyibizi Julius and Nizeyimana Emmanuel revised and finalized the finished manuscript. All authors reviewed the final manuscript before submission.

## Funding

The authors have nothing to report.

## Ethics Statement

The authors have nothing to report.

## Consent

The authors have nothing to report.

## Conflicts of Interest

The authors declare no conflicts of interest.

## Protocols and Registry

This review does not require registry as no protocols were established.

## Data Availability

All relevant data used in this study is provided within the manuscript.
